# Collective Viral Spread Mediated by Virion Aggregates Promotes the Evolution of Defective Interfering Particles

**DOI:** 10.1128/mBio.02156-19

**Published:** 2020-01-07

**Authors:** Iván Andreu-Moreno, Rafael Sanjuán

**Affiliations:** a Institute for Integrative Systems Biology (I^2^SysBio), Universitat de Valencia, Valencia, Spain; Columbia University College of Physicians & Surgeons

**Keywords:** collective infectious units, defective interfering particles, experimental evolution, social evolution, vesicular stomatitis virus

## Abstract

A growing number of studies report that viruses can spread in groups in so-called collective infectious units. By increasing the cellular multiplicity of infection, collective dispersal may allow for social-like interactions, such as cooperation or cheating. Yet, little is known about how such interactions evolve. In previous work with vesicular stomatitis virus, we showed that virion aggregation accelerates early infection stages in most cell types, providing a short-term fitness benefit to the virus. Here, we examine the effects of virion aggregation over several infection cycles. Flow cytometry, deep sequencing, infectivity assays, reverse transcription-quantitative PCR, and electron microscopy revealed that virion aggregation rapidly promotes the emergence of defective interfering particles. Therefore, virion aggregation provides immediate fitness benefits to the virus but incurs fitness costs after a few viral generations. This suggests that an optimal strategy for the virus is to undergo virion aggregation only episodically, for instance, during interhost transmission.

## Abstract (precis)

Recent insights have revealed that viruses use a highly diverse set of strategies to release multiple viral genomes into the same target cells, allowing the emergence of beneficial, but also detrimental, interactions among viruses inside infected cells. This has prompted interest among microbial ecologists and evolutionary biologists in studying how collective dispersal impacts the outcome of viral infections. Here, we have used vesicular stomatitis virus as a model system to study the evolutionary implications of collective dissemination mediated by viral aggregates, since this virus can spontaneously aggregate in the presence of saliva. We find that saliva-driven aggregation has a dual effect on viral fitness; whereas aggregation tends to increase infectivity in the very short term, virion aggregates are highly susceptible to invasion by noncooperative defective variants after a few viral generations.

## INTRODUCTION

Viruses use different strategies for dispersing in groups in so-called collective infectious units (1–3). For instance, some viruses pack multiple genomes inside polyploid capsids to ensure their joint spread (4–6). Other viruses propagate as pools of virions inside extracellular lipid vesicles (7–11) or are embedded in specific proteinaceous structures, such as baculovirus occlusion bodies (12). Viral particles can also form aggregates (13, 14) or attach to the surface of bacteria to undergo joint transmission (15). However, little is known about the evolutionary implications of collective dispersal in viruses.

A common feature among collective dispersal strategies is that they increase the cellular multiplicity of infection (cMOI), defined as the average number of viral genomes that initiates the infection of a cell (16). High cMOIs may increase infectivity by allowing the virus to surmount different types of infection barriers. For instance, initiating the infection with multiple viral genomes could help the virus better counteract cellular innate immunity or could accelerate the infection cycle, thereby keeping the virus ahead of antiviral responses. Additionally, elevating the cMOI could reduce the chances of abortive infections due to stochastic processes occurring during the earliest stages of infection, when low transcription or translation levels, dilution, or degradation of essential components could limit establishment of the infection. These or other possible infection barriers produce an Allee effect at the cellular level, defined as a positive correlation between the per-capita viral progeny production and the cMOI. This Allee effect was demonstrated recently in vesicular stomatitis virus (VSV) and was found to be dependent on cellular permissivity to infection and on the ability of the cell to mount an innate immune response (17). A previous study with HIV-1 also supported the idea that high cMOIs help overcome early barriers to infection (18), and at least three additional studies with influenza A virus (19, 20) and vaccinia virus (21) are consistent with the notion that increasing the cMOI improves infectivity.

Alternatively, elevating the cMOI might increase viral fitness by favoring genetic complementation among deleterious mutants or by promoting other types of beneficial interactions among different variants of the virus. It has been proposed that such diversity-based cooperation should be particularly important in populations of fast-mutating viruses, such as RNA viruses (22–27). Within this framework, experimental results obtained with virion aggregates in poliovirus (14), polyploid capsids in measles virus (28), phosphatidylserine-rich vesicles in enteroviruses (7), and even occlusion bodies in the DNA baculoviruses (29, 30) have been interpreted in terms of cooperation among different viral variants.

On the other hand, an important consequence of high cMOIs is that genetic complementation tends to reduce purifying selection against deleterious mutations, potentially favoring the emergence of cheater-like viruses, such as defective interfering particles (DIPs) (31–33). Social cheaters succeed at the expenses of functional “helper” viral variants by reaping the benefits of cooperation without reciprocating (34). It has been well established that the invasion of viral populations by DIPs reduces average viral population fitness in such a way that may lead the population to extinction (35–37). This being true, and in the absence of mechanisms for avoiding cheater invasion, collective dispersal should be disfavored.

Whether collective dispersal allows for the evolution of cheater viruses or serves as a mechanism for cooperation remains poorly addressed experimentally. Here, we used VSV as a model system for studying the effect of dispersal in aggregates on viral short-term evolution. Our previous results demonstrated that the aggregation of VSV virions confers a short-term fitness benefit to the virus in most cell types by accelerating the viral infection cycle (17). Here, we performed serial transfers of VSV under aggregated versus free-virion spread conditions. We found that virion aggregation rapidly favors the emergence of DIPs. Therefore, aggregation has immediate benefits but incurs costs after a few viral generations.

## RESULTS

### Rapid selection against VSV aggregates.

**FIG 1:**
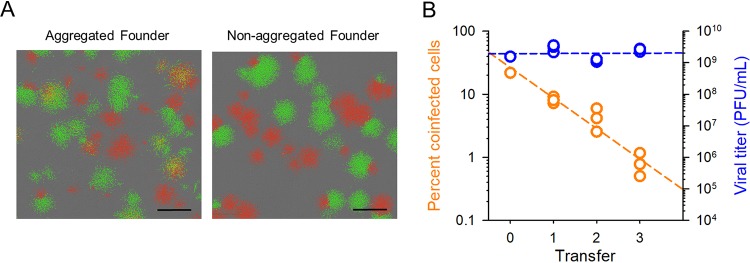
Evolution of saliva-driven coinfection rates. (A) Founder virus. Fluorescence micrographs of infection foci produced at 12 hpi in BHK-21 cells. Right, cells were inoculated with a 1:1 mix of VSV-GFP and VSV-Cherry (nonaggregated). Left, prior to inoculation, viral particles were aggregated in the presence of human saliva. Yellow foci indicate coinfection of cells with the two variants. The gray background shows noninfected cells in phase contrast. Bar = 1 mm. (B) Progressive loss of coinfection in viruses evolved under the saliva-driven aggregation regime (orange) and titer reached by these lines after each transfer (blue). The percentages of cells coinfected with VSV-GFP and VSV-mCherry were determined in cultures infected with saliva-treated virus after each evolution transfer using flow cytometry. Viral titers were quantified after each transfer by the plaque assay. Each dot represents an evolution line (one dot for the founder, three dots for evolved lines). Least-squares regression lines (dashed) are shown.

We used an experimental evolution approach to explore the fitness implications of aggregation in VSV. The evolution was initiated with a 1:1 mix of VSV-green fluorescent protein (VSV-GFP) and VSV-mCherry to track the formation of aggregates, since these tend to produce doubly fluorescent cells. As shown previously (13, 17), VSV virions aggregate in the presence of saliva from certain donors. For the initial virus, we verified that saliva treatment increased the fraction of BHK-21 cells coinfected with green and red variants, indicating an increase in the cMOI, defined as the average number of infectious particles (or, equivalently, genomes for VSV) that initiate the infection of a cell (Fig. 1A). We then performed three serial transfers of the virus, in which viral particles were aggregated in the presence of human saliva before each inoculation. Three evolution replicates were carried out (lines A1, A2, and A3). For these transfers, we used a ratio of 0.01 infectious particles (as determined before aggregation) per cell at inoculation to ensure that coinfection was mainly driven by aggregation (we here use the term “viral density” to refer to the ratio of infectious particles to cells, which should not be confounded with the cMOI). As a control, we performed three evolution lines in which the same experimental protocol was applied, except that virions were not subjected to the aggregation treatment (lines C1, C2, and C3).

After completing the serial transfers, we quantified the ability of the saliva treatment to promote coinfection. For this, we inoculated cells at low viral densities (<0.1 PFU/cell) with saliva-treated viruses and analyzed fluorescence within the first infection cycle by flow cytometry to quantify coinfection events driven by aggregation. Coinfection rates became drastically reduced for viruses evolved under the aggregation regime (0.82% ± 0.19%) compared to the founder virus, whereas control lines evolved without aggregation showed coinfection rates similar to those of the founder (21.2% ± 2.2%; Table 1). An examination of viruses after each transfer showed that the loss of coinfection rates in lines evolved under the aggregation regime was strong but progressive, whereas viral titers remained almost unchanged (Pearson *r =* –0.951; *P < *0.001; Fig. 1B). Hence, after only three serial transfers under saliva-driven aggregation, we largely lost the ability to detect coinfections. In contrast, the titer after each transfer remained approximately constant. We hypothesized two possible explanations for this reduction in coinfection rates. First, selection might have favored virus variants with reduced aggregation capacity. Second, aggregation might have promoted the spread of cheater viruses that interfered with the ability of aggregates to form visible infection foci, leading to lower apparent levels of coinfection.

**TABLE 1:** Percentages of cells coinfected with VSV-GFP and VSV-mCherry in response to saliva treatment for founder and evolved viruses

Virus	% coinfected cells by treatment	
	Saliva treated	Untreated control
Founder	21.9	1.15
Saliva-driven aggregation evolved virus		
A1	0.80	0.53
A2	1.17	0.39
A3	0.50	0.19
Control evolved virus		
C1	18.5	0.99
C2	19.7	1.59
C3	25.5	1.3

### Coinfection loss was probably not driven by reduced virion aggregation.

To test whether the observed loss of cells coinfected with the two fluorescently labeled viruses was due to the emergence of virus variants lacking the ability to aggregate, we first deep sequenced the founder and evolved lines using the Illumina MiSeq platform. If such variants existed, we expected them to map to the envelope glycoprotein G because aggregation ability should in principle be determined by the properties of the virion surface, although other, more indirect mechanisms controlling aggregation cannot be discarded. Two of the lines evolved under the aggregation regime (A2 and A3) shared a haplotype at 12 to 24% frequency containing multiple changes, most of which mapped to the phosphoprotein P and glycoprotein G. However, this haplotype was also found in control line C3 at 16% frequency (Table 2). The mutations that conformed this haplotype were already present in the founder virus population, albeit at much lower frequency (approximately 0.5%; see Table S1 in the supplemental material). Hence, this haplotype was probably favored by selection but does not appear to be related to aggregation capacity because it was found in a control line. Furthermore, many of these mutations, including A1544G, C1622U, U1846C, G2104A, G2925A, A3154G, and A3351G, have been previously reported in other experimental VSV populations (38, 39). Aside from this haplotype, the L892S substitution in the L protein was observed in 10.8% of the reads from line A2 and 1.3% of the reads from line A1. Therefore, we found no evidence for high-frequency genetic variants exclusive to A lines that could explain changes in the aggregation capacities of VSV virions.

**TABLE 2:** Abundances of genetic variants present at >2% frequency in at least one of the evolved populations

Variant	Gene(s)	Mutation	Abundance (%) by virus type^*a*^						
			Founder	A1	A2	A3	C1	C2	C3
G103A	N	V14I	0.63	1.44	5.39	16.45	0.74	0.68	7.11
Haplotype^*b*^	P, M, G	Multiple	ND	ND	11.70	24.04	ND	ND	16.39
A3161G	G		0.32	ND	ND	2.81	0.39	ND	0.20
A3995G	G		4.45	2.02	2.09	2.05	3.24	3.79	3.85
A3999G	G	R308G	4.63	2.07	2.25	1.83	3.50	3.76	4.03
G6372A	L		4.51	3.64	3.50	6.92	4.18	0.94	3.75
U7454C	L	L892S	ND	1.29	10.82	ND	ND	ND	ND
C7458U	L		0.10	2.58	3.54	0.19	ND	0.14	ND
G7729A	L	V984M	1.75	1.57	1.47	1.17	2.20	2.63	1.74
U7966C	L		ND	ND	ND	23.73	ND	ND	ND
U8175C	L		22.37	10.08	12.07	10.60	18.16	18.94	19.00
C8323A	L	L1182I	ND	ND	8.05	ND	ND	ND	ND
A10098G	L		4.77	1.85	2.67	2.19	1.98	2.60	2.28

a A line viruses evolved under aggregation, and C viruses are control lines. ND, not detected above 0.1% frequency.

b Haplotype containing the following linked mutations: U1437C, G1446A, U1524C, A1544G, C1622U, A1632G, A1692C, U1707C, U1740C, C1772U, C1814U, G1833U, U1846C, U1896C, G1899A, G1902A, G1903A, C1961A, A1974C, G2085A, G2104A, C2142U, A2148G, G2221A, C2918U, G2925A, A2949G, U2954C, C2988U, C3003U, A3068C, G3070U, C3071U, U3073A, U3077C, C3101U, U3113C, A3154G, U3182C, U3344C, A3351G, C3491U, U3499C, G3530U, U3591G, U3617C, U3632C, G3719A, G3772A, A3791G, A3938C, A4013C, C4069U, U4070C, C4073U, A4208U, and C4280U.

10.1128/mBio.02156-19.1 TABLE S1 Abundance (% reads) of genetic variants present at >0.1% frequency in at least one of the evolved populations (founder; A line viruses that evolved under aggregation; C, control lines). Download Table S1, CSV file, 0.1 MB. Copyright © 2019 Andreu-Moreno and Sanjuán. 2019 Andreu-Moreno and Sanjuán This content is distributed under the terms of the Creative Commons Attribution 4.0 International license.

**FIG 2:**
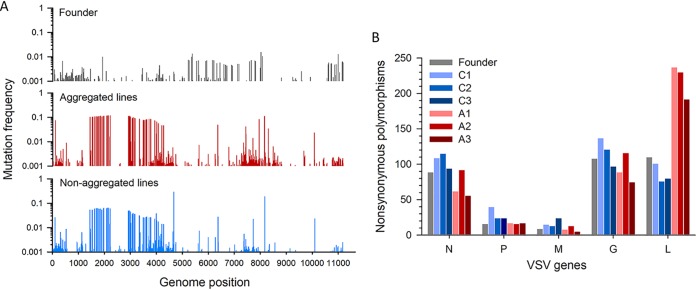
VSV deep sequencing. (A) Nonsynonymous mutation frequencies along the viral genome. Mutation frequencies (no. of mutated reads/no. of total base reads, excluding indels) were calculated by pooling all reads of lineages of the same treatment (aggregated versus nonaggregated). (B) Nonsynonymous polymorphisms at >0.1% in frequency found in VSV genes in the founder virus and evolved lines.

We also analyzed patterns of diversity across the genome. For this, we considered all substitutions present at >0.1% population frequency, even if a fraction of these substitutions may be sequencing errors (Table S1). We obtained gene by gene both the frequency of nonsynonymous mutations in the population and the number of nonsynonymous polymorphic sites. For the N, P, M, and G genes, these diversity measures were not significantly different between lines evolved under aggregation and control lines. In contrast, the L gene displayed both higher mutation frequency (94.3 ± 13.7 versus 20.7 ± 2.7 mutations per million bases; Welch’s *t* test, *P = *0.029; Fig. 2A) and a higher number of nonsynonymous polymorphisms (219.7 ± 14.0 versus 85.7 ± 7.8; Welch’s *t* test, *P = *0.001; Fig. 2B) in lines evolved under aggregation than in control lines. A likely scenario is that these variants of the L gene represented deleterious mutations maintained in the population by genetic complementation at elevated cMOIs.

Overall, the above-described genetic analysis suggests that the observed loss of coinfection rates was not caused by selection favoring viral variants with low aggregation ability. As an alternative explanation for our results, we hypothesized that interfering viruses could be favored by aggregation. This would reduce the ability of virion aggregates to initiate productive infections, hence reducing the number of observable coinfection events.

### Yield reduction assays reveal that aggregation favors the spread of interfering viruses.

**FIG 3:**
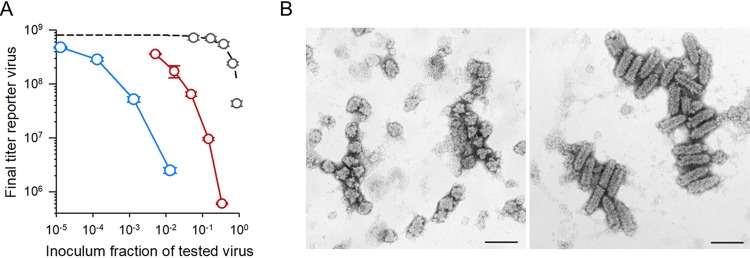
Virion aggregation promotes the emergence of DIPs. (A) Yield reduction assay. The titer of a reporter virus at 16 hpi is shown as a function of the fraction of tested/(reported + tested) viruses in the inoculum. The titer of the reporter virus decayed roughly proportionally to the fraction of founder virus in the inoculum (gray), as expected from direct competition (dashed line; *r^2^* = 0.884). In contrast, the titer of the reported virus decayed faster when mixed with A1 virus (red) or a virus serially transferred at high density (10 PFU/cell; blue), suggesting the presence of interfering viruses in these tested viral populations. (B) Electron micrographs of A3 viruses (left) and C2 viruses (right). Bullet-shaped virions correspond to VSV carrying complete genomes, whereas shorter, thimble-shaped viruses corresponded to DIPs. Scale bars = 200 nm. DIPs were found in all A lines but only rarely in C lines.

We set out to explore whether DIPs or other types of interfering viruses emerged during our serial transfers under the aggregation regime. First, to obtain a DIP-rich population that could be used as a positive control, we used our founder virus to perform three serial transfers in which cells were inoculated at high density (10 PFU/cell). Then, to test for interference, we devised a yield reduction assay. This approach detects interference by quantifying the titer reduction of a reporter virus in the presence of serial dilutions of the samples being tested. Our reporter virus was a monoclonal antibody resistant mutant (MARM), and the samples tested were the founder virus, viruses evolved under the aggregation regime, and our DIP-positive control. At 16 h postinoculation (hpi), we determined the titer produced by the reporter virus by performing plaque assays in the presence of anti-VSV monoclonal antibody. In the presence of the founder virus, the final yield of the reporter virus was proportional to its relative abundance in the inoculum, indicating that the two viruses competed for cellular resources but did not exhibit interference (Fig. 3A). In contrast, the yield of the reporter virus decreased disproportionally with the abundance of line A1 viruses in the inoculum. For instance, adding 5% of line A1 virus to the inoculum reduced the yield of the reporter virus by an order of magnitude, from (8.1 ± 1.2) × 10^8^ to (7.5 ± 0.4) × 10^7^ PFU/ml. Hence, the inoculum interfered with the ability of the reporter virus to produce progeny (or, alternatively, lines evolved under aggregation displayed a strongly increased competitive ability). The positive control showed an even stronger interference, since adding 1% of this virus to the inoculum reduced the yield of the reporter virus by 2 orders of magnitude.

### Aggregation promotes the emergence of defective particles.

Most DIPs lack large portions of the 3′ genome region encompassing the N, P, M, and G genes but retain certain regions of the L gene (40). Thus, we tested the presence of defective genomes by reverse transcription-quantitative PCR (RT-qPCR) using two pairs of primers, one of them mapping toward the end of the L gene (genome positions 9168 to 9367) to quantify total genomes, and another mapping to a region of the P gene (positions 1772 to 1971) to quantify non-DIP genomes. We used the L/P RNA ratio (R) measured by RT-qPCR as an indicator of the abundance of defective genomes. Whereas R was ≈1 for the founder virus, revealing no defective genomes, we obtained an R of >5 for each of the A lines, indicating at least a 4-fold excess of defective genomes (Table 3). The positive control passaged using high viral density (10 PFU/cell) showed an even higher R value, as expected if DIPs became highly abundant. Finally, the three C lines showed R values slightly higher than 1, suggesting a low but detectable frequency of defectives. Although for C lines, each transfer was initiated with a low density, the cMOI probably increased during the final stages of the infection, allowing for the replication of some defective genomes.

**TABLE 3:** RT-qPCR analysis of P and L regions of the VSV genome

Sample	Line	L/P RNA ratio^*a*^	*P* value^*b*^
Founder		1.008 ± 0.035	
Low-virus-density transfers			
	C1	1.751 ± 0.041	0.000
	C2	1.132 ± 0.083	0.272
	C3	1.389 ± 0.094	0.043
Saliva aggregation transfers			
	A1	8.965 ± 0.167	0.000
	A2	5.117 ± 0.343	0.006
	A3	5.407 ± 0.219	0.002
High-virus-density transfers		23.211 ± 3.896	0.029

a Calculated as 2*^CT^*^_P^^–^ *^CT^*^_L^, where *C_T_*_P and *C_T_*_L are the threshold cycle (*C_T_*) values obtained from RT-qPCR of the P and L VSV genome regions, respectively. Three qPCR replicates were performed for each sample. The standard error of the mean (SEM) is shown.

b *t* test against the founder.

To confirm the presence of DIPs, we subjected viruses from the evolved lines and the founder virus to transmission electron microscopy. Whereas the founder virus and control lines evolved in the absence of aggregation showed normal, bullet-shaped virions of approximately 180 by 60 nm, in each of the three A lines, we found shorter capsids exhibiting a typical DIP morphology (Fig. 3B) (40–42).

### Loss of aggregation is reversed following low-cMOI transfers.

To test whether DIPs were responsible for the loss of observable GFP-mCherry VSV coinfections, we performed two additional transfers of the A lines in the absence of saliva-induced aggregation and using a very low viral density at inoculation (<0.001 PFU/cell) to select against DIPs. The resulting viruses and the founder virus were then subjected to saliva-induced aggregation, and GFP-mCherry coinfection rates were measured by flow cytometry, as described above. We found that these further-passaged A lines fully recovered the levels of GFP-mCherry coinfection displayed by the founder virus (45.6% ± 0.4% for the founder virus versus 43.1% ± 1.9% for A lines; *t* test, *P = *0.305; these assays were performed in a different experimental block than those shown on Table 1 and somewhat exhibited higher overall levels of coinfection). We conclude that DIPs were probably responsible for the inability of aggregated virions to produce observable infection foci.

## DISCUSSION

We have found that DIPs tend to accumulate in VSV populations when virions are serially transferred in an aggregated manner. In our assays, DIPs likely participated in virion aggregates and prevented these aggregates from yielding productive infections, reducing the number of observable foci positive for both fluorescently labeled viruses. Alternatively, because DIPs were more abundant than were functional viruses, it is possible that most aggregates contained DIPs exclusively (producing no infection) or contained DIPs and only one of the two fluorescently labeled viruses (producing singly fluorescent foci). Interestingly, the viral titer remained approximately constant despite the emergence of DIPs. A likely explanation for this is that, although saliva promoted aggregation, not all viral particles became aggregated following saliva treatment. Because we used a low viral density at inoculation to start each new infection, a large fraction of infection foci originated from individual particles even in saliva-treated lines. DIPs did not interfere with the formation of these foci; hence, the virus reached roughly normal titers despite the presence of DIPs.

In any case, it seems unlikely that our short-term experimental evolution regime selected for virus variants that failed to aggregate, particularly since deep sequencing revealed no candidate mutations. Furthermore, coinfection rates were restored after two transfers at low inoculation density aimed at removing DIPs. It can be envisaged, though, that longer-term experimental evolution under aggregating conditions might select for viruses capable of avoiding DIP invasion by at least two alternative mechanisms. First, DIP-resistant virus variants could evolve, as previously shown for populations serially transferred at high viral densities (43, 44). Second, mutations leading to a loss of aggregation capacity could be selected, since these would also prevent DIP invasion by reducing the cMOI.

Altogether, these results and our previous findings suggest that VSV aggregation has different implications for viral fitness. In the very short term, increasing the cMOI allows the virus to overcome Allee effects operating at the cellular level, as shown previously (16). These Allee effects might be caused by the presence of early barriers to infection or by stochastic processes acting during early infection stages. Hence, initially, VSV tends to gain a fitness advantage by propagating collectively. Yet, within a few viral generations, increasing the cMOI promotes the emergence of defective viruses, which function as social cheaters and take over the population. Therefore, our results strongly suggest that ongoing virion aggregation during intercellular spread should be evolutionarily disfavored. Given that DIP emergence at high cMOIs is a widely reported process (31–33, 39, 44–47), our results with VSV might as well be valid for other viruses.

In our experiments, potentially any viral particle in the supernatant could aggregate with any other particle present in the same population, regardless of whether they originated from the same cell or from different cells. Theory has established that, in order to avoid cheater invasion, there has to be some factor that increases genetic relatedness among interacting individuals, such as spatial structure or some other sort of assortment among individuals (34, 48, 49). Hence, our experimental results are compatible with the theoretical expectation. In natural VSV infections, saliva-driven aggregation may take place in the oral cavity of infected mammals, which is a preferred viral shedding route for horizontal and vector-borne transmission (50–52). This should allow for mixing between virions produced in different cells (low relatedness). An analogous situation might take place during HIV aggregation in semen, which is induced by prostatic acidic phosphatase amyloid fibrils found in seminal fluid (53). Yet another form of aggregation that may bring together viral particles produced in different cells is attachment of the virus to the surface of intestinal bacterial cells, which has been shown to increase the cMOI and promote recombination in poliovirus (15). If such aggregation occurred frequently, as in our experiments, DIPs should invade the population. However, these forms of aggregation appear to be circumscribed to host-to-host transmission events. As such, they should be episodic and intermingled with multiple cycles of cellular infection during which aggregation may be absent. We speculate that by increasing the cMOI during the very first infection cycles following interhost transmission but not subsequently, aggregation promoted by vehicles such as saliva, semen, or bacteria might help the virus overcome early infection barriers without promoting massive DIP invasion. Interestingly, other types of collective spread can operate during multiple consecutive cell infection cycles, but in these cases, grouping takes place before the virus egresses from cells (for instance, enterovirus vesicles). Collective infectious units formed by viruses produced in the same cell should exhibit high levels of genetic relatedness and hence should be more resistant to DIP invasion.

In previous work, it has been suggested that cooperation among different genetic variants has a positive impact on viral fitness, particularly for fast-mutating RNA viruses (22–27). However, in light of our results, it seems unlikely that virion aggregates, as well as other types of collective infectious units, could support this type of diversity-based cooperation. The reason is that collective viral spread modes that bring together different virus variants should also promote the emergence of cheaters such as DIPs, offsetting the possible benefits of cooperation. Moreover, theoretical work and simulations support the view that genetic complementation among deleterious mutants does not increase mean population fitness over the long term and may even promote error catastrophe (54, 55).

## MATERIALS AND METHODS

### Virus and cells.

Baby hamster kidney fibroblasts (BHK-21; ATCC CCL-10) were cultured in complete Dulbecco’s modified Eagle’s medium (DMEM) supplemented with 10% fetal bovine serum (FBS) at 37°C in a 5% CO_2_ humidified incubator and were mycoplasma free, as determined by PCR. An infectious cDNA clone of the VSV Indiana serotype, originally created by Lawson et al. (56) and kindly provided by Valery Z. Grdzelishvili (University of North Carolina), was used to engineer two VSV variants encoding GFP or mCherry reporters at the intergenic region between the G and L genes. The monoclonal antibody resistance mutant used in yield reduction assays was obtained by passaging VSV-mCherry three times in the presence of anti-G antibody, followed by plaque purification.

### Saliva-driven aggregation.

Saliva-driven aggregation was carried out as described previously (17). Briefly, concentrated viral suspensions (approximately 10^9^ PFU/ml) were diluted 1:10 in human saliva and incubated at 37°C for 1 h before performing convenient serial dilutions for infecting cells.

### Serial transfers.

Confluent BHK-21 monolayers containing approximately 10^7^ cells were inoculated with a 1:1 mix of VSV-GFP and VSV-mCherry with or without aggregation. Inoculation was carried out by incubating cells with virus suspension for 45 min under standard culture conditions (37°C, 5% CO_2_). Cells were overlaid with 1× DMEM supplemented with 2% FBS. Viruses were harvested at 20 to 22 hpi and titrated by the plaque assay to determine the viral titer before initiating the following transfer.

### Plaque assays.

Confluent BHK-21 monolayers were inoculated with serial dilutions of virus suspensions for 45 min under standard culture conditions (37°C, 5% CO_2_). Then, monolayers were overlaid with 1× DMEM supplemented with 2% FBS and 0.6 to 0.7% agar and incubated for 20 to 24 h. Cells were then fixed with 10% formaldehyde, the agar overlay was removed to stain cells with 2% crystal violet in 10% formaldehyde, and plaques were counted.

### Flow cytometry.

BHK-21 cells were inoculated at an approximate density of 0.1 PFU/cell, incubated for 6 h, which, based on the VSV growth dynamics in BHK-21 cells, corresponds to the first infection cycle. Cells were then detached from plates using trypsin-EDTA, resuspended in 1× DMEM containing 10% FBS, washed with 1× PBS by centrifugation (700 × *g*, 5 min), and resuspended in 1 ml of 4% paraformaldehyde for an overnight fixation at 4°C. Then, the fixator was removed and washed with 1× PBS by centrifugation (700 × *g*, 5 min, twice), and cells were resuspended in 1× PBS for analysis in a Becton, Dickinson LSRFortessa flow cytometer equipped with 488- and 561-nm lasers for GFP and mCherry excitation, respectively. Controls containing noninfected cells, singly infected cells (VSV-GFP or VSV-mCherry), and coinfected cells were used to adjust quadrants manually. The percentage of coinfected cells was calculated from approximately 100,000 events.

### Sample preparation for deep sequencing.

Viral RNA was purified using the Quick-RNA viral kit (Zymo Research), following the manufacturer’s instructions. VSV RNA was reverse transcribed and amplified in three overlapping PCR amplicons of approximately 4 kb each, covering the entire VSV genome except for 5′ and 3′ ends used for primer annealing. For each amplicon, a sequence-specific primer was used first for reverse transcription (5′-ACGAAGACAAACAAACCA for amplicon 1, 5′-GGAAAGCATTGAACAAACG for amplicon 2, and 5′-GCTTGCACAGTTCTACTTTC for amplicon 3). Reverse transcriptase (RT) reactions were performed at 42°C with AccuScript Hi-Fi reverse transcriptase (Agilent), following the manufacturer’s instructions. Output cDNAs were subsequently amplified with Phusion high-fidelity DNA polymerase (Thermo Scientific) in 50-μl reaction mixtures containing 3% (vol/vol) dimethyl sulfoxide (DMSO) using the following pairs of primers: amplicon 1, 5′-CCATTATTATCATTAAAAGGCTC and 5′-AGCTAAGATGAAGATCGGAG; amplicon 2, 5′-CTACCACAGAAAGGGAACTG and 5′-GTCTTTAACAAGTTCGCTGG; and amplicon 3, 5′-CAGATCCCGTAACAGAAAGT and 5′-ACGAAGACCACAAAACCAG. The thermal cycling conditions were established as follows: an initial denaturation at 98°C for 1 min, 35 cycles of 98°C for 10 s, 20 s at 56°C for amplicon 1 and 58°C for amplicons 2 and 3, and 72°C for 2 min, followed by 5 min for final extension at 72°C. PCR products were verified by agarose gel electrophoresis, purified with the DNA Clean & Concentrator kit (Zymo Research), and quantified by spectrometry (NanoDrop One; Thermo Scientific). Then, the PCR amplicons of each sample were mixed equimolarly for Illumina sequencing in a MiSeq machine using paired-end libraries.

### Deep-sequencing analysis.

The quality of the reads in FastQ files was evaluated with FastQC 0.11.7 (http://www.bioinformatics.babraham.ac.uk/projects/fastqc/). Then, the first 10 nucleotides and the last two nucleotides of each read were cut with Cutadapt (https://cutadapt.readthedocs.io/en/stable/). Reads were then trimmed using the FASTQ quality filter (http://hannonlab.cshl.edu/fastx_toolkit/) and Prinseq-lite 0.20.4 (57) by quality (>Q30), length (200 nucleotides), and sequencing artifacts (duplications, Ns). The ViVan 0.43 pipeline (58) was used for mapping reads and calling variants using the sequence of our founder cDNA clone as a reference. QuasiRecomb 1.2 was used for haplotype inference (59). Parameters were set to reconstruct only major haplotypes incorporating Phred quality scores. No recombination was assumed since it is computationally intensive and recombination seldom occurs in VSV. In order to hasten the whole-haplotype reconstruction process, BAM files were subsampled for an 8-fold reduction in coverage using SAMtools 1.9, and analyses were performed over four overlapping genomic regions of approximately 3.5 kb.

### Yield reduction assays.

The presence of interfering mutants in samples was assessed by yield reduction assays. VSV-mCherry-MARM was used as the reporter virus and was inoculated into confluent BHK-21 monolayers at 10 PFU/cell, alone or mixed with serial dilutions of the samples to be assayed. After incubating the inoculum for 45 min, cultures were overlaid with 1× DMEM supplemented with 2% FBS and incubated for 16 h under standard culture conditions (37°C, 5% CO_2_). The titer reduction of the reporter virus was quantified by the plaque assay in the presence of a neutralizing monoclonal antibody against the VSV-G protein. This antibody was obtained in-house from a mouse hybridoma cell line, as described previously (60). In the absence of interfering mutants, the final titer of the reporter virus should change proportionally to its abundance in the inoculum. Deviations from this expectation indicated interference.

### RT-qPCR.

Viral RNAs were extracted from culture supernatants using the Quick-RNA viral kit (Zymo Research), following the manufacturer’s instructions. Next, 2 μl of RNA template at a concentration of 10 to 20 ng/μl was used for reverse transcription, which was carried out using AccuScript Hi-Fi RT (Agilent) and gene-specific primers hybridizing to the first half of the P gene (5′-CGCCAGAGGGTTTAAGTGGAG) or to the end of the L gene (5′-AACGATTCCCCACAAGATCCC), following the manufacturer’s instructions. The linear range of detection for the RT reaction was determined using serial dilutions of extracted viral genomes. The qPCR mixtures were loaded with 2 μl of cDNA and the reactions performed using the PowerUp SYBR green master mix (Thermo), with sequence-specific primers, in a QuantStudio 3 machine. Primers for the P gene (5′-CGCCAGAGGGTTTAAGTGGAG and 5′-TTCTGATTGGGACGGATGTGTG) allowed us to determine the number of probably functional genomes, whereas primers for the L gene (5′-AACGATTCCCCACAAGATCCC and 5′-GCAAGAGGGTGGTGGAAATAGAG) allowed us to determine the total number of genomes (functional or DIP). Serial dilutions of a purified plasmid encoding the VSV genome were used to determine and optimize the amplification efficiency for each primer pair. A three-step thermal profile was used for maximum amplification efficiency, as follows: 95°C for 10 min and 40 cycles of 95°C for 5 s, 55°C for 10 s, and 60°C for 20 s. All reactions were run in triplicate, and the absence of primer-dimers and multiple amplicons was tested by melting curve analysis and included no-template controls.

### Transmission electron microscopy.

To obtain viral suspensions sufficiently concentrated for transmission electron microscopy, viruses from evolved lines were amplified by inoculating BHK-21 cells at a viral density of 0.1 PFU/cell. Culture media were harvested after 24 h and were subjected to two serial centrifugations at 3,000 × *g* for 10 min to remove cellular debris. Then, media were centrifuged at 35,000 × *g* for 2.25 h, and pellets were carefully rinsed with 1 ml of PBS. Then, pellets were resuspended in 120 μl of 1× DMEM, centrifuged at 10,000 × *g* for 3 min to further remove small debris, aliquoted, and stored at –70°C. These preparations were mixed 1:5 for A lines and 1:10 for C lines with 1× phosphate buffer (PB), and 20 μl was mixed 1:1 with 4% paraformaldehyde (PFA) and 5% glutaraldehyde fixator diluted in 1× PB and incubated for 1 h at room temperature. Then, 5 μl per sample was carefully placed on Formvar carbon-coated grids and air dried for at least 1 h before rinsing three times with Milli-Q water filtered through 0.2-μm cellulose filters. Finally, samples were stained with 2% phosphotungstic acid for less than a minute, dried with filter paper, and observed under a transmission electron microscope.
